# Sensitive and rapid detection of cholera toxin-producing *Vibrio cholerae *using a loop-mediated isothermal amplification

**DOI:** 10.1186/1471-2180-8-94

**Published:** 2008-06-12

**Authors:** Wataru Yamazaki, Kazuko Seto, Masumi Taguchi, Masanori Ishibashi, Kiyoshi Inoue

**Affiliations:** 1 Division of Bacteriology, Osaka Prefectural Institute of Public Health, Osaka, Japan

## Abstract

**Background:**

*Vibrio cholerae *is widely acknowledged as one of the most important waterborne pathogen causing gastrointestinal disorders. Cholera toxin (CT) is a major virulence determinant of *V. cholerae*. Detection of CT-producing *V. cholerae *using conventional culture-, biochemical- and immunological-based assays is time-consuming and laborious, requiring more than three days. Thus, we developed a novel and highly specific loop-mediated isothermal amplification (LAMP) assay for the sensitive and rapid detection of cholera toxin (CT)-producing *Vibrio cholerae*.

**Results:**

The assay provided markedly more sensitive and rapid detection of CT-producing *V. cholerae *strains than conventional biochemical and PCR assays. The assay correctly identified 34 CT-producing *V. cholerae *strains, but did not detect 13 CT non-producing *V. cholerae *and 53 non-*V. cholerae *strains. Sensitivity of the LAMP assay for direct detection of CT-producing *V. cholerae *in spiked human feces was 7.8 × 10^2 ^CFU per g (1.4 CFU per reaction). The sensitivity of the LAMP assay was 10-fold more sensitive than that of the conventional PCR assay. The LAMP assay for detection of CT-producing *V. cholerae *required less than 35 min with a single colony on thiosulfate citrate bile salt sucrose (TCBS) agar and 70 min with human feces from the beginning of DNA extraction to final determination.

**Conclusion:**

The LAMP assay is a sensitive, rapid and simple tool for the detection of CT-producing *V. cholerae *and will be useful in facilitating the early diagnosis of human *V. cholerae *infection.

## Background

*Vibrio cholerae *is widely acknowledged as one of the most important waterborne pathogen causing gastrointestinal disorders. Cholera toxin (CT) is a major virulence determinant of *V. cholerae*. This bacterium is indigenous to fresh and blackish water environments in tropical, subtropical and temperate areas worldwide, the threat of epidemic cholera is restricted primarily to developing countries with warm climates [1-3]. *V. cholerae *causes seafood borne infection, water-borne outbreaks and epidemics in terrestrial environments [1,3]. Therefore, ingestion of raw or undercooked seafood such as shrimp and drinking water contaminated with *V. cholera *are risk factors in humans [1-3]. Most *V. cholerae *isolates from the environment do not produce CT, nor do they possess the genetic potential to produce CT. *V. cholerae *O1 and O139 are the major seroytpes associated with illness, and some *V. cholerae *non-O1 and non-O139 isolates produce CT. These findings necessitate regular examination of *V. cholerae *isolates for their ability to produce CT in order to assess their clinical significance [3,4].

Detection of CT-producing *V. cholerae *using conventional culture-, biochemical- and immunological-based assays is time-consuming and laborious, requiring more than three days. Commercially available kits can not distinguish between the heat-labile enterotoxin (LT) of *Escherichia coli *and CT. A rapid, reliable and practical assay for the detection of CT-producing *V. cholerae *has thus been sought. Several PCR assays offer a more sophisticated approach to the identification of *Vibrio cholerae *[4,5]. Although PCR assays provide more rapid identification of *Vibrio cholerae *than conventional assays, they require the use of electrophoresis to detect amplified products, which is time-consuming and tedious. Real time PCR assays recently developed for the rapid identification of *Vibrio cholerae *[2,6] are not routinely used due to their requirement for an expensive thermal cycler with a fluorescence detector.

Among other techniques, however, one promising candidate is a novel nucleic acid amplification method termed loop-mediated isothermal amplification (LAMP) [7]. LAMP is based on the principle of autocycling strand displacement DNA synthesis performed by the *Bst *DNA polymerase large fragment for the detection of a specific DNA sequence with specific characteristics [8]. This offers a number of advantages: first, all reactions can be carried out under isothermal conditions ranging from 60 to 65°C; second, its use of six primers recognizing eight distinct regions on the target nucleotides means that specificity is extremely high [9]; and third, detection is simplified by visual assessment using the unaided eye, without the need for electrophoresis [10,11]. Thus, LAMP assay is faster and easier to perform than conventional PCR assays, as well as being more specific [12,13]. Furthermore, because the LAMP assay synthesizes a large amount of DNA, the products can be detected by simple turbidity. Thus, compared to PCR assays, expensive equipment is not necessary to give a high level of precision [10,12,13]. These features allow simple, rapid and cost-effective detection [13,14]. Also, the increase in the turbidity of the reaction mixture according to the production of precipitate correlates with the amount of DNA synthesized [10,11]. Various LAMP assays for the identification of pathogenic organisms have been developed [10-13,15,16], however, no assay for the detection of CT-producing *V. cholerae *has been described.

Here, we describe a sensitive, rapid and simple LAMP assay for the detection of CT-producing *Vibrio cholerae*. Sensitivity was determined in pure cultures and spiked human feces.

## Results

**Figure 1:**
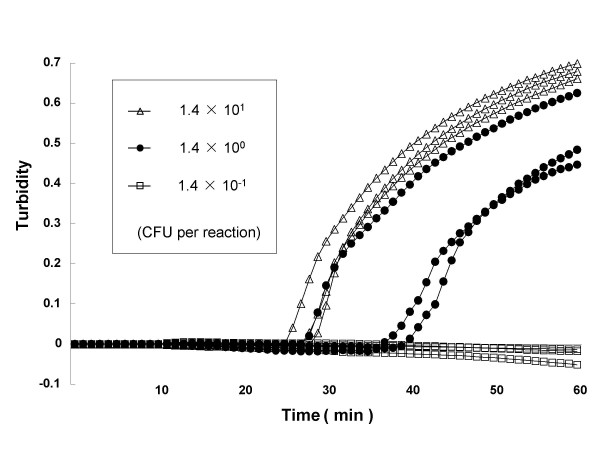
**Sensitivity test for detection of CT-producing *V. cholerae *from spiked human feces by real-time turbidimetry**. The curves from left to right indicate decreasing concentrations of CFU from bacterial colonies [1.4^1 ^to 1.4^-1^CFU per reaction].

LAMP products were detected from all 34 CT-producing *V. cholerae *strains. No LAMP products were detected from any of the 13 CT non-producing *V. cholerae *and 53 non-*V. cholerae *strains (Table 1). The PCR assay required more than 4 h, while the LAMP assay was markedly faster, requiring for amplification 12–18 min with a single colony on TCBS agar from each of 34 CT-producing *V. cholerae *strains and less than 45 min with spiked human feces (Fig. 1). The assay required less than 35 min and 70 min for detection of CT-producing *V. cholerae *with a colony on TCBS agar and with spiked human feces from the beginning of DNA extraction to final determination.

**Table 1:** Results of the LAMP assay

Species	Strains	No. of strains	LAMP results	Production of CT/LT	Source
*Vibrio cholerae *O1		26			
	61H151		+	+	Human feces, Japan?, 1986
	4H77		+	+	Human feces, Thailand, 1992
	5H176		+	+	Human feces, Indonesia, 1993
	5H332		+	+	Human feces, Indonesia, 1993
	6H58		+	+	Human feces, Thailand, 1994
	6H62		+	+	Human feces, Unknown, 1994
	6H346		+	+	Human feces, Indonesia, 1994
	7H164		+	+	Human feces, Indonesia, 1995
	7H285		+	+	Human feces, Japan, 1995
	8H215		+	+	Human feces, Japan, 1996
	9H3		+	+	Human feces, Thailand, 1997
	10H1		+	+	Human feces, China, 1998
	10H169		+	+	Human feces, Philippines, 1998
	10H664		+	+	Human feces, Philippines, 1999
	11H215		+	+	Human feces, India, 1999
	11H558		+	+	Human feces, Madagascar, 2000
	13H59		+	+	Human feces, Indonesia, 2001
	13H82		+	+	Human feces, Japan, 2001
	13H173		+	+	Human feces, Japan, 2001
	13H257		+	+	Human feces, Indonesia, 2001
	15H245		+	+	Human feces, Thailand, 2004
	17H16		+	+	Human feces, Indonesia, 2005
	18H24		+	+	Human feces, India, 2006
	62H92		-	-	Human feces, Japan?, 1987
	2H283		-	-	Human feces, Indonesia, 1991
	11H259		-	-	Human feces, Egypt/Greece, 1999
*Vibrio cholerae *O139		13			
	236-93		+	+	Human, India, 1993
	1034-93		+	+	Human, Pakistan, 1993
	183-93		+	+	Human, Bangladesh, 1993
	21-93		+	+	Human, Chennai, India, 1993
	65-93		+	+	Human, Kolkata, India, 1993
	481-93		+	+	Human, Thailand, 1993
	495-96		+	+	Human, Nepal, 1996
	99-93		+	+	Human, Vellore, India, 1993
	147-93		+	+	Human, Madurai, India, 1993
	VC-23		+	+	Unknown, 1995
	333-93		+	+	Pond, India, 1993
	1033-93		-	-	Human, Sri Lanka, 1993
	Arg-3		-	-	Human, Argentine
*Vibrio cholerae *non-O1/non-O139		8			
	61H37		-	-	Human feces, Thailand, 1986
	3H264		-	-	Human feces, Indonesia, 1992
	5H231		-	-	Human feces, Indonesia, 1993
	9H237		-	-	Human feces, China, 1997
	9H300		-	-	Human feces, India, 1997
	12H207		-	-	Human feces, 2000
	19H149		-	-	Human feces, 2007
	3H222		-	-	Clam, Japan, 1991
LT-producing *E. coli*		7	- (0/7)	+ (7/7)	Human feces, Japan
*V. parahaemolyticus*		6	-	ND	Human feces, Japan
*V. vulnificus*		3	-	ND	IFO15645^T ^and human feces, Japan
*V. alginolyticus*		2	-	ND	IFO15630^T ^and unknown source
*V. fluvialis*		1	-	ND	Human feces
*V. furnissii*		1	-	ND	Human feces
*V. harveyi*		1	-	ND	IFO15634^T^
*V. metschnikovii*		1	-	ND	Human feces
*V. mimicus*		1	-	ND	Human feces
Other bacteria		30	-	ND	Described in Methods.

+, positive reaction; -, negative reaction (positive number/strain number tested); ND, not determined.

As shown in Table 2, sensitivities of the LAMP assay for CT-producing *V. cholerae *O1 strain 13H173 with pure cultures and spiked human feces were found to be 7.8 × 10^2 ^CFU per ml (2.9 CFU per reaction) and 7.8 × 10^2 ^CFU per g (1.4 CFU per reaction). Further, the sensitivity of the LAMP assay was 10-fold higher than that of the PCR assay (Table 2). The dilutions of 10^-3^-10^-4 ^(14.4 – 1.4 CFU per reaction) showed an increase in turbidity (Fig. 1) and was visible as white turbidity but not that of 10^-5 ^(0.1 CFU per reaction). Sensitivities determined by the two methods were constantly matched with each other.

**Table 2:** Sensitivity of the LAMP assay for CT-producing *V. cholerae*

Strain	Specimens		Dilutions of cultures for the assays			
			
			10^-2^	10^-3^	10^-4^	10^-5^
CT-producing *V. cholerae*						
13H173	Pure cultures	CFU per reaction	288.8	28.8	2.9	0.3
		LAMP	+	+	+	± (2/3)
		PCR	+	+	± (1/3)	-
	Spiked human feces	CFU per reaction	144.4	14.4	1.4	0.1
		LAMP	+	+	+	-
		PCR	+	+	-	-

+, triplicate assay showed all positive.

±, triplicate assay showed both positive and negative (positive number/tested number).

-, triplicate assay showed all negative.

## Discussion

The bacterial culture test for the isolation and identification of CT-producing *V. cholerae *from human feces required 3–4 d, with plating onto selective agars, sequential subculture and CT productivity test. In contrast, the LAMP assay was markedly faster. For PCR assay, 4–5 h is required for amplification, electrophoresis and staining, while the LAMP assay requires for DNA extraction from specimens and amplification less than 35–70 min. Further, amplification of the LAMP assay could be judged by visual assessment using the unaided eye, without the need for electrophoresis. The LAMP assay was more sensitive, rapid and simple than the conventional PCR assay. Therefore, the LAMP assay is more effective in detecting CT-producing *V. cholerae *than the conventional PCR assay.

CT is closely related to LT at the immunological and genetic levels, [4], therefore their discernment is critical. A commercial reversed passive latex agglutination assay kit for the detection of CT/LT is available. However, this kit is unable to discern between CT and LT. Although PCR assays have been shown suitable for the specific detection of the *ctx *gene without confusing the *lt *gene [4,5], the procedure is time-consuming and tedious. We therefore developed a new and specific LAMP assay for CT-producing *V. cholerae*. A primer set based on the *ctxA *gene was designed to prevent the confusion of CT and LT with highly conserved and specific regions for CT.

The sensitivity of the LAMP assay shown in Table 2 seems a little high, and Table 2 indicates detection of 0.3 CFU per reaction in 2/3 replicates. We adopted 6 h-enrichment not to reach stationary phase for the determination of the sensitivity, according to Fedio *et al *[17]. However, the samples may, to some extent, contain DNAs derived from dead or viable but non-cultivable (VNC) cells [18] in the present study, which may have affected the sensitivity we determined. Further work is needed to confirm this hypothesis.

The frequent outbreaks caused by CT-producing *V. cholerae *in developing countries [1,3] highlight the need for the rapid and accurate identification of the species. We successfully developed the first LAMP assay for detection of CT-producing *V. cholerae *from spiked human feces. Application of this assay to food and environmental microbiology should facilitate a comprehensive approach to the control of cholera infection and the rapid and sensitive detection of small numbers of CT-producing *V. cholerae *in food and environmental specimens.

## Conclusion

The LAMP assay provided markedly more sensitive, simple and rapid detection of CT-producing *V. cholerae *than conventional biochemical and PCR assays. Further, it can be applied to the direct detection of CT-producing *V. cholerae *with spiked human feces. The LAMP assay for detection of CT-producing *V. cholerae *required less than 35 min with a colony on TCBS agar and 70 min with spiked human feces from the beginning of DNA extraction to final determination. The LAMP assay is a powerful tool for the rapid and sensitive detection of CT-producing *V. cholerae*, and will facilitate the early diagnosis of cholera in humans.

## Methods

### Bacterial strains

A total of 100 bacterial strains were used, including 34 CT-producing *Vibrio cholerae *strains, as well as an additional 13 CT non-producing *Vibrio cholerae *and 53 non-*Vibrio cholerae *strains as reference strains and field isolates. The 47 *Vibrio cholerae *strains are detailed below, and also shown in Table 1. Twenty-six O1, thirteen O139 and eight non-O1/non-O139 *Vibrio cholerae *strains were obtained from clinical patients of overseas travelers and domestic cases, and a food specimen between 1986 and 2007 in Japan. Fifteen non-*Vibrio cholerae *reference strains were obtained from international culture collections (*Arcobacter butzleri *ATCC49616^T ^(American Type Culture Collection, USA); *Arcobacter cryaerophilus *ATCC43158^T^; *Arcobacter skirrowii *ATCC51132^T^; *Campylobacter coli *JCM2529^T ^(Japan Collection of Microorganisms, Saitama, Japan); *Campylobacter fetus *subsp. *fetus *ATCC27374^T^; *Campylobacter jejuni *subsp. *jejuni *LMG8841^T ^(Culture Collection of the Laboratorium voor Microbiologie, University of Ghent, Belgium); *Campylobacter lari *JCM2530^T^; *Campylobacter upsaliensis *ATCC43954^T^; *Escherichia coli *ATCC25922, and ATCC35218; *Pseudomonas aeruginosa *ATCC27853; *Staphylococcus aureus *subsp. *aureus *ATCC25923;*Vibrio alginolyticus *IFO15630^T ^(Institute for Fermentation, Osaka, Japan); *Vibrio harveyi *IFO15634^T^; and *Vibrio vulnificus *IFO15645^T^). A superscript T designates a type-strain. Thirteen non-*V. cholerae Vibrio *strains were obtained from clinical patients or unknown sources, as follows: 6 *V. parahaemolyticus*, 2 *V. vulnificus*; and one strain each of *V. alginolyticus*, *V. fluvialis*, *V. furnissii*, *V. metschnikovii*, and *V. mimicus*. Twenty-five non-*Vibrio *strains were obtained from clinical sources, as follows: seven heat-labile enterotoxin (LT)-producing *Escherichia coli *strains (O25:HNM, O159:H2, O159:H27, O167:HUT, O169:H41, OUT:H12, OUT:HUT); five LT non-producing *Escherichia coli*; and one strain each of *Aeromonas hydrophila*, *Aeromonas sobria*, *Citrobacter freundii*,*Enterobacter cloacae*, *Helicobacter pylori*, *Klebsiella pneumoniae*, *Morganella morganii*, *Plesiomonas shigelloides*, *Proteus mirabilis*,*Providensia alcalifaciens*, *Salmonella enterica *serovar Enteritidis, *Shigella flexneri *1a, and *Shigella sonnei*.

### Storage and culture conditions

All *Vibrio *strains were stored in Casitone semi-solid broth (Eiken Chemical Co., Ltd., Tokyo, Japan) or cooked meat broth (Becton Dickinson and Co., Sparks, MD, USA) at room temperature until required. They were grown on thiosulfate citrate bile salt sucrose agar (TCBS agar; Eiken Chemical) and incubated overnight at 35°C. All *Arcobacter*, *Campylobacter *and *Helicobacter *strains were stored in brucella broth (Becton Dickinson) containing 10% (v/v) horse serum and 10% (v/v) DMSO at -80°C, until required. They were grown on blood agar supplemented with 5% (v/v) lysed horse blood, and incubated for 2–3 days in a microaerobic atmosphere, except *H. pylori*, which was incubated for 10 days. Incubation was at 37°C except *A. cryaerophilus*, which was grown at 30°C. Other bacterial strains were stored in cooked meat broth at room temperature until required, and grown on blood agar or tryptic soy agar (TSA; Nissui, Tokyo, Japan) and cultured overnight at 37°C. CT/LT productivities of *V. cholerae *and *E. coli *strains were determined by a reversed passive latex agglutination assay kit (VET-RPLA; Denka Seiken, Tokyo, Japan) following a manufacturer's instruction.

### DNA extraction from culture

Bacterial DNA was extracted as previously described [19] with slight modification. A single loopful of culture on TCBS agar, blood agar or TSA was inoculated in 50 μl of NaOH (25 mmol l^-1^) in a 1.5-ml microcentrifuge tube using a disposable loop (1-mm diameter), and the cell mixture was heated at 95°C for 5 min. After neutralization with 4 μl of Tris-HCl buffer (1 mol l^-1^), cell debris was pelleted by centrifugation at 20,000 *g*, 4°C, for 5 min and the supernatant was used as template DNA for the LAMP assay.

### LAMP assay

**Figure 2:**
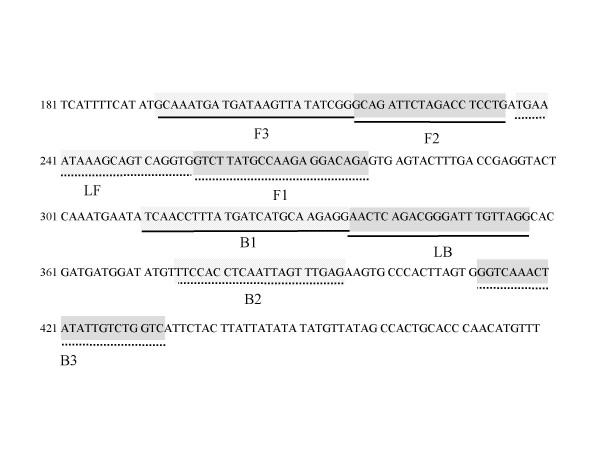
**Locations of the target sequences used as primers**. The name and location of each target sequence as a primer in *ctxA *gene of *V. cholerae *K02679.

LAMP assay was performed with a Loopamp DNA amplification kit (Eiken Chemical). The final LAMP assay comprised 2 μl of template DNA, 1 μl of *Bst *DNA Polymerase (Eiken Chemical), 1.6 μmol l^-1 ^each of inner primers FIP and BIP, 0.2 μmol l^-1 ^each of outer primers F3 and B3, and 0.8 μmol l^-1 ^each of loop primers LoopF and LoopB, in a 1 × Reaction Mix (Eiken Chemical). Final volume was adjusted to 25 μl. All primers were produced by Hokkaido System Science Co., Ltd. (Sapporo, Japan), and designed from sequence data submitted to GenBank (Cholera toxin subunitA gene, *ctxA*, K02679) [20] with Primer Explorer V4 software (Fujitsu System Solutions Ltd., Tokyo, Japan). To find specific nucleotide sequences of CT-producing *V. cholerae*, a multiple alignment was determined with analyses of 34 *ctxA *sequences (AE003852, AF175708, AF390572, AF414369, AF452584, AF463400–AF463401, AF510994–AF510998, AF516341–AF516349, AF542088–AF542089, AJ575590, AY101181, CP000626-CP000627, D30052–D30053, DQ774432, K02679, X00171, X58785–X58786) from DDBJ/EMBL/GenBank data base. The sequences and locations of each primer are shown in Table 3 and Fig. 2. Primer FIP consisted of the F1 complementary sequence and the F2 sequence. Primer BIP consisted of the B1 sequence and the B2 complementary sequence. Primer B3 and LF consisted of the B3 and LF complementary sequences, respectively. The mixture was incubated at 65°C for 60 min and then at 80°C for 2 min to terminate the reaction in a Loopamp real-time turbidimeter (LA-320; Teramecs, Kyoto, Japan). LAMP amplification was detected as a value of turbidity at 650 nm using a LA-320 in real-time. The reaction was considered to be positive when the turbidity reached 0.1 within 60 min. Turbidity visible with the unaided eye was also considered to indicate a successful LAMP procedure.

**Table 3:** LAMP primers used

GenBank accession no.	Primer	Sequence (5' to 3')	Gene location (bp)
K02679	CtxA-FIP	TCT GTC CTC TTG GCA TAA GAC GCA GAT TCT AGA CCT CCT G (F1c-F2)	277-257 (F1c), 217–235(F2)
	CtxA-BIP	TCA ACC TTT ATG ATC ATG CAA GAG GCT CAA ACT AAT TGA GGT GGA A (B1-B2c)	311–335(B1), 395-375(B2c)
	CtxA-F3	GCA AAT GAT GAT AAG TTA TAT CGG (F3)	193–216
	CtxA-B3	GMC CAG ACA ATA TAG TTT GAC C (B3c)	433-412
	CtxA-LF	CAC CTG ACT GCT TTA TTT CA (LFc)	256-237
	CtxA-LB	AAC TCA GAC GGG ATT TGT TAG G (LB)	336–357

All primers were designed from the sequence of *ctxA *gene of *V. cholerae *K02679, submitted to GenBank by Lockman *et al*., 1984 [20].

### Determinations of sensitivities of the LAMP assay with pure cultures and spiked human feces

The sensitivities of the LAMP assay for the detection of CT-producing *Vibrio cholerae *with pure culture and spiked human feces were determined as previously described [11] with slight modification using known amounts of *Vibrio cholerae *O1 strain 13H173 (Table 1). A single culture on TCBS agar was inoculated in alkaline peptone water (APW; Eiken chemical) and incubated at 35°C for 6 h. Serial 10-fold dilutions of the culture were prepared in PB (Phosphate buffer). For preparation of DNAs from pure cultures, 100 μl of each was transferred to a 1.5-ml microcentrifuge tube, and was centrifuged for 5 min at 20,000 g. After removal of the supernatant, the pellets were resuspended in 50 μl of NaOH (25 mmol l^-1^), and the mixture was heated at 95°C, for 5 min. After neutralization with 4 μl of Tris-HCl buffer (1 mol l^-1^, pH 7.5), debris was pelleted by centrifugation at 20,000 g, 4°C, for 5 min. For preparation of DNAs from spiked human feces, 100 μl of each was spiked into 100 mg of a *V. cholerae*-negative human feces. The fecal sample was obtained from a *Norovirus*-positive patient with diarrhoea. The fecal sample was determined to be negative for *V. cholerae *according to the results of a microbiological examination with overnight APW enrichments and subsequent plating onto TCBS agar. The fecal homogenates were then adjusted to a 10% concentration with PB. After mixing well, the homogenate was centrifuged at 900 g for 1 min to remove larger fecal debris. Supernatant was transferred to a new 1.5-ml microcentrifuge tube, and was centrifuged for 5 min at 10,000 g. After removal of the supernatant, the pellets were resuspended in 100 μl of NaOH (25 mmol l^-1^), and the mixture was heated at 95°C, for 5 min. After neutralization with 8 μl of Tris-HCl buffer (1 mol l^-1^, pH 7.5), debris was pelleted by centrifugation at 20,000 g, 4°C, for 5 min. Two microliters of each supernatant was then used as template DNA for LAMP assay. The sensitivity tests of the LAMP assay were conducted in triplicate, and the detection limits were defined as the last positive dilutions, with the sample considered positive if all three samples tested positive. In parallel, to enumerate the bacteria, 100-μl aliquots of appropriate dilutions were spread on Heart Infusion agar (Becton Dickinson) and incubated overnight at 35°C. Colonies were counted at the dilution yielding 30 to 300 Colony Forming Units (CFUs), and CFU per ml/g of suspension was calculated.

### PCR assay

A multiplex PCR assay targeting *ctxA*, O1-*rfb *and O139-*rfb *genes [5] was performed in a 50-μl reaction mixture containing 2 μl of template DNA and the respective primer (Hokkaido System Science Co., Ltd.) in 1 × Qiagen Multiplex PCR Master Mix (Qiagen GmbH, Hilden, Germany). The sequences of primers were as described in published papers [5]. The concentrations of all primers were adjusted 0.2 μM. DNA amplification was performed in a TaKaRa PCR Thermal Cycler Dice Gradient (TaKaRa Bio, Otsu, Japan). The cycling conditions used were one cycle of 95°C for 15 min, 35 cycles each of 94°C for 1 min, 55°C for 1.5 min and 72°C for 1 min, and ending with a final extension time at 72°C for 7 min. Samples were held at 4°C prior to analysis. PCR products were subjected to electrophoresis in 2% agarose gels. After staining with ethidium bromide, the PCR products were detected under UV light. The sensitivity of the PCR assay was determined using template DNA from pure cultures and spiked cells in human feces as described above. The sensitivity tests of the PCR assays were conducted in triplicate, and the detection limits were defined as the last positive dilutions, with the sample considered positive if all three samples tested positive.

## Authors' contributions

WY carried out LAMP and PCR assays; KS and MT isolated and identified bacterial strains together; WY and MI conceived the study; KI coordinated the study. All authors read and approved the final manuscript.
